# Surface Plasmon Resonance Microscopy Based on Total Internal Reflection

**DOI:** 10.3390/bios13020261

**Published:** 2023-02-12

**Authors:** Teliang Zhang, Xueliang Wang, Youjun Zeng, Songfeng Huang, Xiaoqi Dai, Weifu Kong, Qian Liu, Jiajie Chen, Junle Qu, Yonghong Shao

**Affiliations:** 1 Key Laboratory of Optoelectronic Devices and Systems of Ministry of Education and Guangdong Province, College of Physics and Optoelectronic Engineering, Shenzhen University, Shenzhen 518060, China; 2 College of Physics and Optoelectronic Engineering, Guangdong University of Technology, Guangzhou 510006, China

**Keywords:** total internal reflection, surface plasmon resonance, surface plasmon resonance microscopy, biomolecular interaction

## Abstract

Surface plasmon resonance microscopy (SPRM) has been widely employed in biological fields because of its high spatial resolution and label-free detection modality. In this study, SPRM based on total internal reflection (TIR) is studied via a home-built SPRM system, and the principle of imaging of a single nanoparticle is analyzed as well. By designing a ring filter and combining it with the deconvolution algorithm in Fourier space, the parabolic tail of the nanoparticle image is removed, in which a spatial resolution of 248 nm is obtained. In addition, we also measured the specific binding between the human IgG antigen and goat anti-human IgG antibody using the TIR-based SPRM. The experimental results have proved that the system can image sparse nanoparticles and monitor biomolecular interactions.

## 1. Introduction

Surface plasmons (SPs) are collections of electrons oscillating and propagating along the surface of a metal. Under certain conditions, a surface plasmon wave (SPW) is produced as the incident light interacts with free electrons near the metal/dielectric interface. When the incident wave vector satisfies the wave vector’s matching condition, the energy of the incident light is converted to that of the SPW in large quantities, resulting in a decrease in the energy of the reflected light, which is called surface plasmon resonance (SPR) [1]. Therefore, SPR sensing systems can monitor the binding process of surface molecules by detecting alterations in the SPR position of the sample, and researchers can obtain important information, such as the binding and dissociation constants by analyzing the binding process profile [2]. To achieve high-throughput detection, SPR has been combined with imaging technology to develop a high-throughput, high-sensitivity SPR imaging sensing system (SPRi) [3,4]. Conventional SPRi has low spatial resolution and cannot detect single molecules or nanoparticles; therefore, SPR microscopy (SPRM) was developed to address this challenge. Two typical SPRM systems have been developed, i.e., prism-based SPRM [4] and objective-based SPRM [5]. Prism-based SPRM has higher sensitivity and throughput. Our group has proposed wavelength-scanning surface plasmon resonance microscopy (WS-SPRM), which serves as a label-free biosensor capable of measuring cell-substrate interactions [6]. We also combined the optothermophoretic flipping method with SPRM and actively drive the aggregation of protein molecules toward the sensing surface, which significantly improves the sensitivity [7].

However, prism-based SPRM is limited by the working distance of the objective lens and imaging distortion, and it is difficult to distinguish the dynamic process of molecules or nanoparticles in the submicron region. Objective-based SPRM has the characteristics of high spatial resolution, which is more suitable for exploring the dynamic process of sparse particles [8]. For example, Tao et al. built an oil-immersion objective SPRM based on a commercial microscope and studied cell-matrix interactions by detecting alterations in SPR intensity [9]. Karan et al. further investigated antimicrobial susceptibility testing [10]. Aaron et al. monitored the interaction of DNA molecules carrying nanoparticle balls in real time [11]. Yang et al. report an optical method for imaging and size analysis of exosomes using interferometric plasma microscopy (iPM) [12]. Yu et al. further analyzed individual DNA molecules quantitatively [13] and achieved high-resolution imaging of nanoparticles using a semicircle-like filter utilized in the frequency domain and a deconvolution algorithm [14].

**Figure 1:**
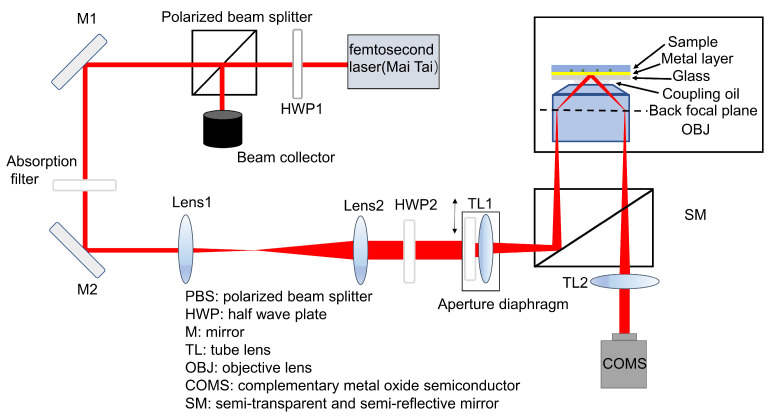
Schematic of total internal reflection-surface plasmon resonance microscopy.

In this study, total internal reflection-based surface plasmon resonance microscopy (TIR-SPRM) is developed. The TIR-SPRM system uses a wavelength-adjustable femtosecond laser as the light source. The femtosecond light source has excellent parallelism and a broader spectrum range so that the speckles induced by the light interference can be minimized. Therefore, we can obtain high quality SPR images, and a three-dimensional displacement stage is implemented to adjust the incident angle via the movement of the TL1 and diaphragm (Figure 1). Therefore, the angle and wavelength can be optimized simultaneously, and thus the point of maximum absorption can be found without knowing the specific refractive index of the sample. The ability of the system to monitor biomolecular interactions at the microscopic scale is verified by detecting the specific binding of the human IgG and goat anti-human IgG antibodies. In addition, the imaging resolution of 248 nm without SPR-induced imaging distortion is achieved, in which the parabolic tailing in the nanoparticle image is removed by a designed ring filter and deconvolution algorithm at the Fourier domain.

## 2. Materials and Methods

### 2.1. Instrumentation

The TIR-SPRM experimental setup is illustrated in Figure 1, where a femtosecond laser (Mai Tai HP, Spectral Physics) with a central wavelength of 720 nm is utilized as the excitation light source. The power of this laser is adjusted by an optical attenuator, which is combined with a polarized beam splitter and a half-wave plate 1 (HWP1). The laser is deflected by reflectors 1 (M1) and 2 (M2), and the deflected light is expanded by a 16×beam expansion system comprising lenses 1 (f = 25 mm) and 2 (f = 400 mm). Half-wave plate 2 (HWP2) is utilized to set the polarization state of the incident light to P-polarized light. Tube lens 1 (TL1, f = 200 mm) and the aperture diaphragm form a three-dimensional moving device to adjust the angle of incidence. The incident light converges to the back focal plane of the high-magnification objective (100×, NA = 1.49), which is turned into a parallel beam on the surface of the gold film. The light scattered by the object on the surface of the gold film is imaged to the complementary metal oxide semiconductor (CMOS) via the high-magnification objective and built-in tube lens (TL2, f = 200 mm) of the Nikon microscope. Simultaneously, molecular interactions on the surface of the gold film cause changes in the refractive index at the interface between the gold film and solution, resulting in changes in the reflected light intensity received by the COMS, thereby monitoring the molecular interaction process.

### 2.2. SPR Sensing Principle

In our SPRM system, an objective-based coupling method is adopted, and its principle is illustrated in the box area of Figure 1. Coupling oil with a refractive index of 1.515 is used. It is filled between the gold-coated glass substrate and objective lens to achieve the refractive index matching, and the gold film thickness is 48 nm. The sample to be tested is placed on the upper layer of the gold film. The incident light passes through the objective lens and excites the gold film at the resonance angle, where the wave vectors of the incident light and SPW are matched, and SPR occurs.

When the SPR phenomenon occurs, the relationship between the wave vector of the SPW and incident light can be expressed as:

(1) $$k_{\mathrm{spw}}=\frac{k_{i}}{n_{o}}sin\theta_{R},$$

where $k_{\mathrm{spw}}$ is the wave vector of the SPW, $k_{i}$ is the wave vector of the incident light, $n_{o}$ is the refractive index of the optically dense medium, and $\theta_{R}$ is the resonance angle. Moreover, $k_{\mathrm{spw}}$ can be expressed as

(2) $$k_{\mathrm{spw}}=\frac{2\pi }{\lambda }\sqrt{\frac{\varepsilon_{1}\varepsilon_{m}}{\varepsilon_{1}+\varepsilon_{m}}},$$

where λ is the wavelength of the incident light, $\varepsilon_{m}$ is the permittivity of the metal [15], and $\varepsilon_{1}$ is the dielectric constant of the medium.

When the wavelength of incident light is fixed, $\theta_{R}$ can be expressed as:

(3) $$sin\theta_{R}=\sqrt{\frac{\varepsilon_{1}\varepsilon_{m}}{\left(\varepsilon_{1}+\varepsilon_{m}\right)\varepsilon_{o}}},$$

where $\varepsilon_{o}$ is the dielectric constant of the glass substrate. According to Equation (3), the change in the dielectric constant of the medium changes the resonance angle $\theta_{R}$ as well, and the change in $\theta_{R}$ changes the intensity of the reflected light. Therefore, molecular interactions between samples with high coverage and weak scattering can be characterized by detecting changes in light intensity.

### 2.3. Scattering Principle of Nanoparticles and Image Reconstruction

**Figure 2:**
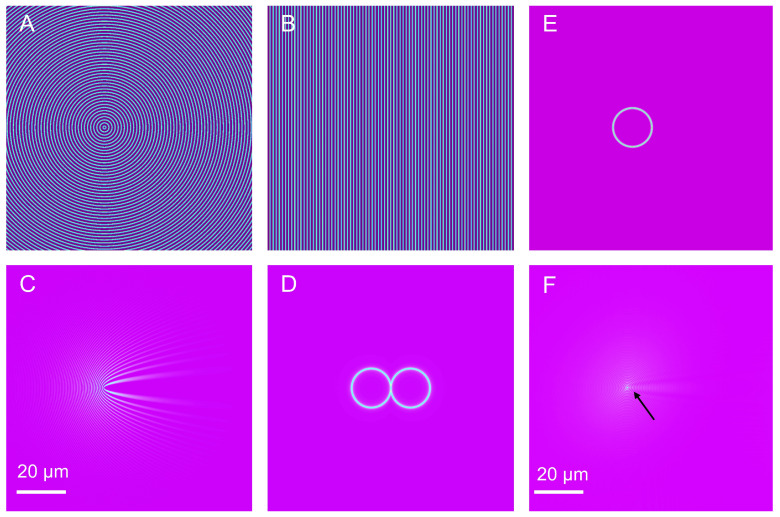
Simulation of SPRM imaging and reconstruction process for nanoparticle. (**A**) Scattered wave of nanoparticle; (**B**) SPW propagated at interface between gold film and medium; (**C**) SPRM image of nanoparticle and two-dimensional Fourier transform; (**D**,**E**) filter *F*_1_; (**F**) reconstruction result for the nanoparticle. The black arrow points to the simulated nanoparticle deconvolution results.

In TIR-SPRM, where strong scattering objects (e.g., nanoparticles) are placed near the metal surface, the SPRM image exhibits a parabolic diffraction pattern. The SPRM imaging process of nanoparticles [16] includes the scattering of nanoparticles and SPW transmission, as illustrated in Figure 2A,B. The scattering wave of the object interferes with the SPW, resulting in an SPRM image with a parabolic shape, as illustrated in Figure 2C.

The SPRM image I of nanoparticle is expressed as follows [15]:

(4) $$I=|E_{sp}+E_{s}{|}^{2}=\left|E_{sp}{|}^{2}+\right|E_{s}{|}^{2}+E_{SP}^{*}\cdot E_{s}+E_{sp}\cdot E_{s}^{*}$$

where $E_{sp}$ is the SPW electric field, and $E_{s}$ is the scattering wave electric field produced by nanoparticles excited by SPW. The first, second, and third represent plane wave, scattered wave, and interference superposition electric field, respectively, and the fourth term represents the conjugate of the third term.

The parabolic shape of the SPRM image needs to be removed because it significantly affects the spatial resolution of the SPRM. Although removing the parabolic shape in the spatial domain is difficult, a deconvolution algorithm [17] can be adopted to remove it effectively in the frequency domain.

The two-dimensional Fourier transform of image I comprises two intersecting circles, as illustrated in Figure 2D, which contain information about the interference superposition field. Here, based on Yu’s semicircular ring-like filter [14], a simplified circular ring filter $F_{1}$ is constructed to obtain the scattered wave electric field, as illustrated in Figure 2E. $F_{1}$ can be expressed as follows:

(5) $$F_{1}\left(k_{x},k_{y}\right)=\exp^{-\left(\sqrt{{\left(k_{x}-k_{x1}\right)}^{2}+{\left(k_{y}-k_{y1}\right)}^{2}}-k_{r}^{2}\right)/(2k_{1}{)}^{2}},$$

where $\left(k_{x1},k_{y1}\right)$ is the center position of the ring, $k_{r}$ is the radius of the ring, and $k_{1}$ is the width of the ring. The position of $\left(k_{x1},k_{y1}\right)$ and value of $k_{r}$ are determined by the wave vector $k_{\mathrm{spw}}$ where the SPR phenomenon occurs [18,19]. The size of $k_{1}$ can be obtained by fitting the Hendrik Lorentz function [20]. The system point spread function $P_{\mathrm{SF}}$ is adopted to deconvolute with $E_{s}$ filtered by $F_{1}$, which is expressed as:

(6) $$F\left\{O\right\}=F\left\{E_{S}\right\}/F\left\{P_{\mathrm{SF}}\right\},$$

where F{O} and $F\left\{E_{S}\right\}$ are the two-dimensional Fourier transforms of the nanoparticle image and scattering wave electric field of the nanoparticle, respectively. $P_{\mathrm{SF}}$ is the point spread function of the system. $F\left\{P_{\mathrm{SF}}\right\}$ is the two-dimensional Fourier transform of $P_{\mathrm{SF}}$ and a single-ring spectrum filtered by $F_{1}$.

As the noise of $E_{S}$ is slightly magnified owing to the small value of $F\left\{P_{\mathrm{SF}}\right\}$, $F_{1}$ is utilized to filter F{O} again, leading to a reconstructed image of the nanoparticles, as illustrated in Figure 2F.

## 3. Results and Discussion

To verify the ability of TIR-SPRM to monitor the interaction between molecules, a biomolecular interaction experiment was designed. First, the surface of the chip with the gold film was successively cleaned with deionized water and absolute ethanol and blown dry with nitrogen. After these operations, tiny molecules on the surface of the chip were removed, thereby preventing interference with the experimental results. Subsequently, the chip with the gold film was packaged to create a microfluidic channel, and the antigen was immobilized on the gold membrane surface by physical adsorption method, and the experimental steps were as follows.

**Figure 3:**
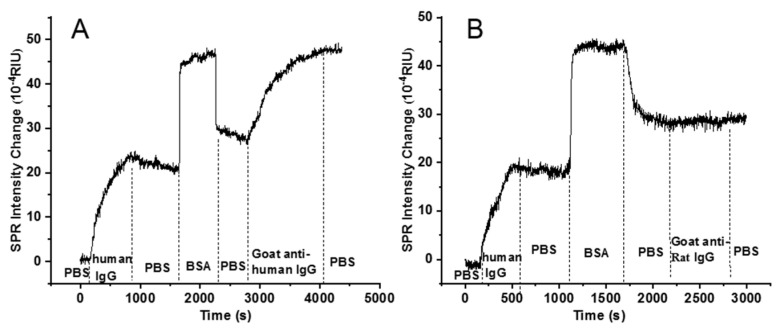
The curve of binding process. (**A**) Human IgG antigen and goat anti-human IgG antibody; (**B**) human IgG antigen and the goat anti-rat IgG antibody.

First, phosphate-buffered saline (PBS, 0.01 M, PH = 7.4) was injected into the microfluidic channel, and its SPR signal intensity change was monitored in real time. When its SPR signal intensity was stable, 20 μg/mL of human IgG antigen was injected. When human IgG was stably bound to the sensing surface of the gold membrane and its SPR signal intensity became stable, the weakly attached human IgG antigen was washed away by injecting PBS. After the SPR signal intensity became stable, 10 mg/mL bovine serum albumin was injected to occupy the empty sites on the chip to prevent the antibodies from binding to the empty sites on the chip method. Similarly, when the SPR signal intensity stabilized, PBS was injected and the free BSA was washed away. After the above steps, goat anti-human IgG antibody of 15 ug/mL was passed to specifically bind to the modified human IgG antigen on the surface of the chip, and after its SPR signal intensity was smooth, PBS was injected to wash away the free goat anti-human IgG antibody. Specific SPR response curves are illustrated in Figure 3A. The refractive index unit (RIU) of human IgG antigen changed by 2.20 × 10^−3^ during fixation on the surface of the gold film. When BSA was injected, the RIU changed by 2.53 × 10^−3^. The reason for the jump in the curve was that the concentration of BSA was much greater than that of the human IgG antigens. When the goat anti-human IgG antibody was injected, the RIU changed by 1.98 × 10^−3^.

To verify the specificity in the human IgG antigen and the goat anti-human IgG antibody interaction experiment, we added a control experiment between 20 μg/mL of human IgG antigen and 15 μg/mL of the goat anti-rat IgG antibody, and the procedure of the control experiment is identical to the human IgG and the goat anti-human IgG antibody experiment. The results are shown in Figure 3B, which shows that when 20 μg/mL human IgG was injected, the RIU changed by 2.20 × 10^−3^. When 10 mg/mL of BSA was utilized to block redundant sites on the chip surface, the RIU changed by 2.86 × 10^−3^. When 15 μg/mL of the goat anti-rat IgG antibody was introduced, the RIU did not change, which indicates that human IgG did not bind to the goat anti-rat IgG antibody, thus proving that the system has the ability to detect specific binding between biomolecules.

**Figure 4:**
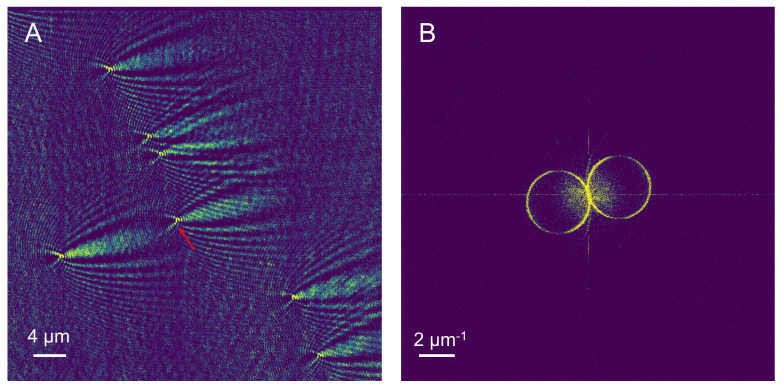
SPRM imaging for 200 nm polystyrene nanospheres; (**A**) and their two-dimensional Fourier transform (**B**).

To verify the high-resolution imaging performance of TIR-SPRM, a solution with 200 nm polystyrene nanospheres was injected into the microfluidic chip. The solvent of in the 200 nm polystyrene nanosphere solution was PBS which is the buffer used for biomolecule binding experiment so that the overall solution refractive index is identical to the biosensing experiment. Furthermore, CMOS was utilized at a speed of 96 f/s for 10 s to obtain SPRM images of the SRRM background and 200 nm polystyrene nanospheres, respectively. The background noise of the system was further removed by time subtraction [19], and the original SPRM image of the obtained polystyrene nanospheres is illustrated in Figure 4A. A two-dimensional Fourier transform was then performed, as illustrated in Figure 4A, and the result is illustrated in Figure 4B, in which the spectrum of the two rings contains the coherent superposition spectrum of the 200 nm polystyrene nanospheres.

**Figure 5:**
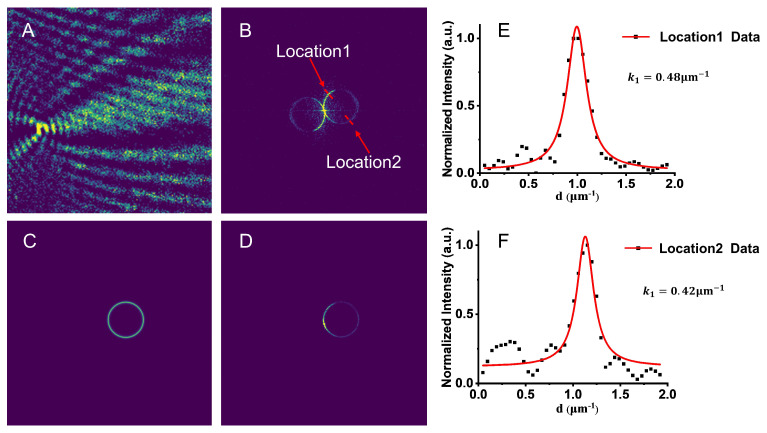
Original SPRM image of a single 200 nm polystyrene nanosphere and its filter in frequency domain. (**A**) SPRM image of a single 200 nm polystyrene nanosphere; (**B**) two-dimensional Fourier transform of (**A**); (**C**) ring filter; (**D**) the spectrum obtained by applying the ring filter (**C**) to (**B**); (**E**,**F**) intensity curves along the red dotted line at the Location 1 and Location 2 on the right ring in (**B**); d represents the spatial frequency.

The PSF of the TIR-SPRM system was derived from a single 200 nm polystyrene nanosphere indicated by the arrow in Figure 4A, and the result is shown in Figure 5B. Subsequently, a two-dimensional Fourier transform was performed, and the results are illustrated in Figure 5B. The intensity values of the dotted line on the left circle in Figure 5B were extracted and plotted as curves, as illustrated in Figure 5E,F. The black points represent the actual intensity value of the ring along the dotted line, and the red curve is the fitted Lorentzian function. By fitting the curve, the ring width $k_{1}$ in the two directions in Figure 5B was calculated as 0.48 μm^−1^ and 0.42 μm^−1^, respectively. The slight difference in the ring widths in these two directions was affected by the focal depth of the objective lens, and a larger $k_{1}$ was chosen because more information could be obtained.

The other parameters of $F_{1}$ were calculated by substituting the center wavelength of 720 nm, the refractive index of PBS solution of 1.338, and metal/dielectric constant into the formula (1), and the calculated results were: $k_{\mathrm{spw}}=1.96\;{\mu m}^{-1}$, $\left(k_{x1},k_{y1}\right)$ = $\left(0.50\;{\mu m}^{-1},\;1.89\;{\mu m}^{-1}\right)$ and $k_{r}=1.96\;{\mu m}^{-1}$. The center position of the circle and values of $k_{r}$ and $k_{1}$ were substituted into Equation (5) to construct $F_{1}$, and the result is illustrated in Figure 5C. The spectrum of the object containing only the scattered field was then obtained by filtering Figure 5B using Figure 5C, and the result is illustrated in Figure 5D.

**Figure 6:**
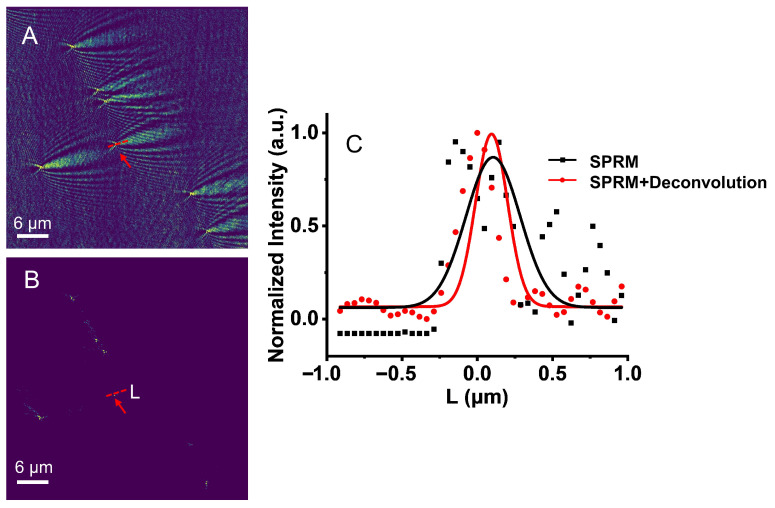
Reconstruction and analysis for SPRM imaging of 200 nm polystyrene nanospheres. (**A**) SPRM image of 200 nm polystyrene nanospheres; (**B**) reconstruction images; (**C**) intensity distribution in L direction.

The filter illustrated in Figure 5C was applied to filter Figure 4B to obtain the filtered spectrum. Furthermore, the filtered spectrum was deconvoluted, and the 200 nm polystyrene nanospheres image was reconstructed, as illustrated in Figure 6B. The intensity curves for the polystyrene nanospheres in the L direction marked by arrows in the original SPRM image in Figure 6A and the reconstructed image in Figure 6B were obtained, as illustrated in Figure 6C. In the L direction, the full widths at half maximum (FWHM) of the polystyrene nanospheres before and after reconstruction were 416 nm and 248 nm, respectively, demonstrating that the resolution of SPRM was increased by 1.68 fold.

In order to better demonstrate the SPR imaging and biosensing ability, we also conducted a transferrin biomolecular interaction experiment. First, 30 mg/mL of transferrin antibody 2 carrying 100 nm polystyrene particles (PS) and 20 μg/mL of transferrin antigen were mixed at a concentration of 1:100 and stirred for 30 min, hereinafter referred to as mixture-PS.

Then, phosphate-buffered saline (PBS, 0.01 M, PH = 7.4) was injected into the microfluidic channel, and its SPR signal intensity change was monitored in real time. When its SPR signal intensity was stable, 20 μg/mL of transferrin antibody 1 as ligand was injected. When transferrin antibody 1 was stably bound to the sensing surface of the gold membrane and its SPR signal intensity became stable, the weakly attached transferrin antibody 1 was washed away by injecting PBS. After the SPR signal intensity became stable, 10 mg/mL bovine serum albumin was injected to occupy the empty sites on the chip to prevent the anti-bodies from binding to the empty sites on the chip method. Similarly, when the SPR signal intensity stabilized, PBS was injected and the free BSA was washed away. Subsequently, the mixture-PS was injected as the analyte to specifically bind to the modified transferrin antibody 1 on the surface of the chip, and after its SPR signal intensity was stable, PBS was injected to wash away the unbonded analyte.

**Figure 7:**
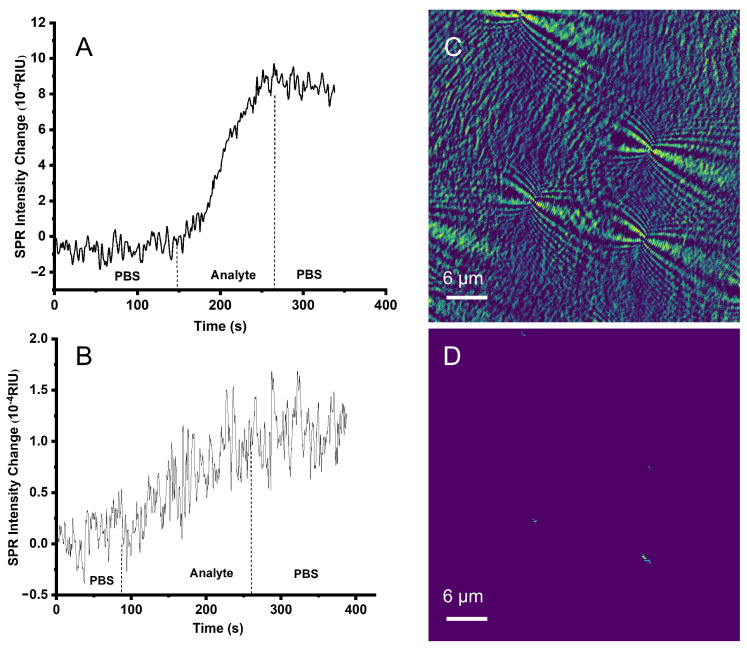
Transferrin-specific binding experiment. (**A**) Binding curve of mixture-PS and transferrin antibody 1; (**B**) binding curve of mixture-noPS and transferrin antibody 1 in the control group; (**C**) SPRM image during the binding process; and (**D**) reconstruction image of (**C**).

The specific binding curve of analyte and ligand is shown in Figure 7A; when 15 μg/mL of mixture-PS (analyte) was introduced as specific binding, the change in RIU was 8.57 × 10^−4^. Figure 7C was an image collected by the system when the mixture-PS specifically binds to the ligand, and the image was filtered and deconvoluted to obtain the result of Figure 7D. To illustrate the signal amplification effect of nanoparticle spheres, we added a set of control experiments, and the control experimental ligand was still 20 μg/mL transferrin antibody 1. Additionally, 30 mg/mL transferrin antibody 2 without 100 nm polystyrene particles was mixed with 20 ug/mL of transferrin antigen at a concentration of 1:100 and left for 30 min, hereinafter referred to as mixture-noPS. The specific binding curve of analyte to mixture-noPS is shown in Figure 7B; when 15 μg/mL of mixture mixture-noPS was introduced as specific binding, the change in RIU was 1.06 × 10^−4^. By comparing Figure 7A with Figure 7B, the nanoparticles amplify the protein signal by 8 times, which proves that the nanoparticles have a signal amplification effect on protein-specific experiments.

## 4. Conclusions

We have developed a TIR-SPRM technique and achieved high-resolution nanoparticle image reconstruction using our simplified circular ring filter, addressing the challenge of the parabolic smearing of nanoparticle images. Biomolecule binding experiments of human IgG and goat anti-human IgG antibodies have verified that TIR-SPRM is capable of biomolecule interaction detection. Its high-resolution imaging capability was verified by imaging nanoparticles and reconstructing the nanoparticle image. Finally, the combination of nanoparticles and protein experiments verified that nanoparticles can amplify the signal of proteins and improve the sensitivity of the system. Compared with our previous study of prism-based SPR microscopy in the Kretschmann configuration [6], the microscopy imaging ability is greatly enhanced, and an imaging resolution of ~200 nm is achieved. As a trade-off, because of the shrinking of the image field of view and the imaging algorithm, the sensitivity of the TIR-SPRM technique becomes lower, while one can enhance it with the assistance of nanoparticles. Therefore, this TIR-SPRM technique is more suitable for the study of more exquisite bio-experiments at the single-molecule level. In the future, the filter will be optimized to further improve the resolution of SPRM. Simultaneously, we will try to visualize the procedures and the dynamics of biomolecule interactions at ultra-low concentrations down to the single-molecule level.

## Data Availability

No new data were created or analyzed in this study. Data sharing does not apply to this article. We used only publicly available datasets for experimentation.
